# Machine Learning-Based Sensor Data Fusion for Animal Monitoring: Scoping Review

**DOI:** 10.3390/s23125732

**Published:** 2023-06-20

**Authors:** Carlos Alberto Aguilar-Lazcano, Ismael Edrein Espinosa-Curiel, Jorge Alberto Ríos-Martínez, Francisco Alejandro Madera-Ramírez, Humberto Pérez-Espinosa

**Affiliations:** 1 CICESE-UT3, Tepic 63173, Mexico; 2 Computer Science Department, Faculty of Mathematics, Autonomous University of Yucatan, Merida 97000, Mexico

**Keywords:** sensor, animals, animal computer interaction, machine learning, sensor fusion

## Abstract

The development of technology, such as the Internet of Things and artificial intelligence, has significantly advanced many fields of study. Animal research is no exception, as these technologies have enabled data collection through various sensing devices. Advanced computer systems equipped with artificial intelligence capabilities can process these data, allowing researchers to identify significant behaviors related to the detection of illnesses, discerning the emotional state of the animals, and even recognizing individual animal identities. This review includes articles in the English language published between 2011 and 2022. A total of 263 articles were retrieved, and after applying inclusion criteria, only 23 were deemed eligible for analysis. Sensor fusion algorithms were categorized into three levels: Raw or low (26%), Feature or medium (39%), and Decision or high (34%). Most articles focused on posture and activity detection, and the target species were primarily cows (32%) and horses (12%) in the three levels of fusion. The accelerometer was present at all levels. The findings indicate that the study of sensor fusion applied to animals is still in its early stages and has yet to be fully explored. There is an opportunity to research the use of sensor fusion for combining movement data with biometric sensors to develop animal welfare applications. Overall, the integration of sensor fusion and machine learning algorithms can provide a more in-depth understanding of animal behavior and contribute to better animal welfare, production efficiency, and conservation efforts.

## 1. Introduction

### 1.1. Multi-Modal Animal Monitoring

Technological advancements, including sensors, the Internet of Things (IoT), machine learning (ML), and big data, have revolutionized the development of intelligent monitoring applications in various domains [1,2,3,4,5]. These transformative technologies have also found extensive application in animal monitoring, serving diverse objectives such as economic and production interests, animal welfare and care, and scientific research on different species, ranging from livestock to wildlife [6].Sensor technologies offer both challenges and opportunities for animal farmers to achieve various goals, such as reducing production costs, improving operational efficiencies, enhancing animal welfare, and maximizing animal yield per hectare [7]. Additionally, researchers are actively exploring innovative noninvasive techniques to promote animal well-being, moving away from invasive approaches [8].To model animal behavior, it is essential to collect a substantial amount of diverse data sets that encompass a wide range of information, including local weather data, air quality data, animal vocalizations, visual recordings of animal movements, and other relevant behavioral data. Researchers can efficiently capture real-time data using various sensors, ensuring minimal disruption to the animals’ daily routines. Data collection must be facilitated through an automated system designed to integrate with the everyday lives of animals seamlessly. In the context of animal monitoring, it is often necessary to automate the identification of specific patterns within collected sensor data, especially when dealing with large datasets. Although specific factors such as temperature changes, movement levels, or heart rate can be easily detected using a single sensor. Certain aspects pose more significant challenges, such as determining animal activity, detecting signs of pathological behavior, or identifying shifts in mood. The complex nature of these aspects suggests that developing effective recognition methods relies on utilizing multiple modalities or employing multiple sensors of the same modality whose information complements one another [9].

### 1.2. Sensor Fusion

Sensor fusion is a powerful method in computer engineering and signal processing to combine information from multiple sensors and generate a more accurate and comprehensive output. This technique draws inspiration from the human sensory system and finds applications in various domains, including robotics, autonomous vehicles, and surveillance systems. In the realm of animal computer interfaces, sensor fusion plays a crucial role in integrating data from various sensors to gain a deeper understanding of the animal’s activities, posture, health, and behaviors. The combination of information from multiple sensors to overcome individual sensor limitations is referred to as multimodal sensor fusion. This is achieved through different fusion techniques [10], such as single fusion algorithm, Unimodal switching, Multimodal switching, and Mixing, to name a few. The single fusion algorithm involves the raw data or the extraction of features from each individual sensor modality and then incorporating all these extracted features into a unified fusion algorithm. Unimodal switching refers to a method where a single mode is utilized to identify changes in operating modes and switch between different sensor fusion techniques. The second mode is exclusively used as the input for each of the sensor algorithms. Multimodal switching involves using one mode to detect shifts in operating modes and switch between various sensor fusion algorithms. Each of the sensor algorithms employs multiple modes as inputs. Mixing refers to the simultaneous operation of numerous sensor fusion algorithms integrating one or more modes. The results from these algorithms are combined with the weight of each output being determined based on a single mode.

### 1.3. Sensor Fusion in Animal Monitoring

In animal monitoring, the use of multiple sensors enables researchers to obtain more accurate and comprehensive data. By combining different sensors, researchers can achieve a holistic understanding of the behavior and health of the animal. For instance, a camera captures visual information regarding the animal’s movements and behavior, an accelerometer provides insights into the animal’s activity level, and a heart rate monitor offers data on the animal’s physiological state. However, the raw signals acquired from these sensors must undergo processing, analysis, and interpretation to become valuable to stakeholders. Sensor fusion techniques take advantage of complementary, redundant, and cooperative attributes to enhance data interpretation. Traditional sensor fusion techniques include probabilistic fusion, evidence-based belief fusion, and rough set-based fusion. However, with advances in sensor technology, processing hardware, and other data processing technologies, new opportunities arise for data fusion [11]. Sensor fusion techniques can be classified into cooperative, competitive, and complementary systems based on low, feature, and high levels. This hierarchical organization, proposed by Elmenreich [12], offers a systematic approach to understanding and designing sensor fusion systems. This categorization helps to identify technical requirements and limitations. In the modeling process, different strategies can be employed to merge information from sensors [8], such as early fusion (sensor-level combination), intermediate fusion (feature-level combination), and late fusion (decision-level combination). These sensor fusion techniques enhance the accuracy and reliability of monitoring systems.

### 1.4. Machine Learning and Sensor Fusion in Animal Monitoring

Machine learning techniques are vital for enhancing data fusion algorithms by capitalizing on the abundance of available data to train models and achieve high-precision results. Machine learning empowers computers to learn from data examples without explicit programming, enabling flexibility and adaptability in handling complex tasks. Prominent techniques involve training models with labeled datasets and automating new data classification. With the rise in machine learning, impressive progress has been made in image classification and object detection, motivating researchers to apply artificial intelligence for action recognition of animals, such as mice, cows, pigs, Tibetan antelope, and felines, among others [13]. In addition, wearable devices based on the Inertial Measurement Unit (IMU) sensor device are becoming more prevalent. It can be used, for example, to support the automatic behavior classification of grazing sheep and the evaluation of production performance [14,15]. There are also promising applications for the interaction between different animals, search and rescue missions, and the protection of animals from poaching and theft [7].

The evaluation performed by Leoni et al. [16] in terms of the precision of the prediction and the interpretability of the decision-making process shows that multimodal systems can overcome the challenges of a hostile environment, which proves to be an effective support for intelligent remote automatic profiling. Integration of multiple sensors, sensor fusion, and machine learning techniques holds great potential to revolutionize our understanding and management of animals. This integration can improve animal welfare, production efficiency, and conservation efforts. For example, in animal farming, the successful application of these technologies has facilitated the production of more meat and animal products, which benefits farmers and the industry as a whole [17]. In agriculture, automated recognition of behavior patterns in livestock can lead to the early identification and resolution of health or welfare issues, resulting in healthier and more productive animals [18]. In the field of conservation biology, the automated recognition of patterns in wild animals offers valuable insights into their behavior and movements, enabling the development of effective conservation strategies [19]. Using the power of artificial intelligence, machine learning, and sensor fusion, we can unlock significant advancements in animal-related domains, fostering improvements in various sectors and driving positive impacts. In recent years, there has been an increased social emphasis on the treatment of animals in research. As a result, when using sensor fusion and machine learning techniques in animal research, it is essential to prioritize animal welfare. This involves implementing measures to provide appropriate care, ensure comfortable conditions, and employ human practices throughout the research process. Ethical guidelines often require researchers to obtain approval from ethics committees or institutional review boards, which evaluate proposed research protocols to ensure compliance with ethical standards. By adhering to these ethical guidelines, researchers can demonstrate their commitment to responsible and ethical practices in animal research. This promotes animal welfare and helps to build public trust and confidence in the scientific community.

### 1.5. Workflow of Machine Learning-Based Sensor Fusion

**Figure 1:**
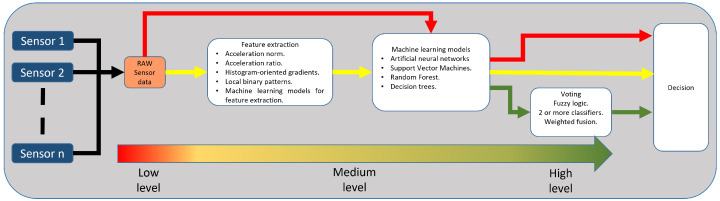
Representation of the three levels of sensor fusion.

In order to perform sensor fusion integrating machine learning techniques, it is necessary to collect data from sensors and process it according to the required level of sensor fusion. Figure 1 illustrates the general workflow of sensor fusion at each level. The low-level fusion, also known as raw level, uses information from multiple sources without prior data processing. ML models are fed with these data to get answers. This can be observed in Figure 1 following the red line. The second level is the intermediate or feature level. During this phase, the data from multiple sources are reduced in dimensionality before being combined by an ML model. This example can be seen by following the yellow line of Figure 1. The third level determines the result by combining one or more algorithms during the high or decision level. The combination can be decided through methods such as majority vote, statistical models, or fuzzy logic. This is shown in Figure 1 following the green line.

## 2. Objectives

Sensor fusion systems for animal monitoring have gained attention in computer, behavioral, and veterinary sciences. Research in this field aims to enhance animal monitoring through sensor fusion applications. Understanding current approaches, implementations, and evaluation methods is crucial for advancing this area. Previous reviews have covered topics such as wearable sensors for animal health management [20], precision livestock farming technologies [21], and the integration of sensor technologies and machine learning in animal farming [22]. However, there is a gap in investigating machine learning-based sensor fusion techniques in animal monitoring. This review aims to bridge this gap by comprehensively examining existing literature and discussing recent advancements in sensor fusion and machine learning for animal monitoring. This review addresses the following six key questions:

1. What specific challenges or problems have been addressed through the application of machine learning-based sensor fusion techniques in the field of animal monitoring?
2. Which animal species have been the primary subjects of studies exploring the application of machine learning-based sensor fusion techniques in the field of animal monitoring?
3. What are the applications of machine learning-based sensor fusion systems for animal monitoring?
4. What sensing technologies are utilized in machine learning-based sensor fusion applications for animal monitoring?
5. How have machine learning-based fusion techniques been utilized for animal monitoring in sensor fusion applications?
6. What are the documented performance metrics and achieved results in machine learning-based sensor fusion applications for animal monitoring?

This review aims to provide valuable insights to technologists developing intelligent animal monitoring solutions, researchers in animal behavior seeking automated data capture and analysis, and computer scientists working on information fusion for animal monitoring. This review aims to contribute to the progress and development of intelligent animal monitoring systems by conducting a comprehensive analysis.

## 3. Methods

We followed a scoping review methodology to synthesize research on the current application of sensor fusion through machine learning in the field of animal monitoring. This protocol followed the PRISMA-ScR (Preferred Reporting Items for Systematic Reviews and Meta-Analyses extension for Scoping Reviews) methodology [23] to ensure that our review was conducted systematically and was bias-free.

### 3.1. Eligibility Criteria

To conduct this scoping review, we focused on studies in English of at least four pages in length, published between 2011 and 2022, that met the following criteria: (1) focus on the description of sensor fusion, (2) sensor fusion is based on machine learning techniques, and (3) focus on animal monitoring.

### 3.2. Exclusion Criteria

To evaluate every document included in this review, we performed a quantitative analysis using the QualSyst standard [24], which provides a structured approach to ensure the quality of the reviewed articles. This analysis includes fourteen evaluation points, of which thirteen related to the research design were selected. These points can be consulted in Table 1. The Qualsyst standard for each of the assessing points can be assigned as follows: two points (completely met), one point (partially met), or zero points (not met). The total score is divided by the number of assessment points. This score is expressed as a percentage from 0% to 100%. We excluded studies with a Qualsyst score <60 %.

### 3.3. Information Sources

We conducted a comprehensive search for published papers using four databases, including the Institute of Electrical and Electronics Engineers (IEEE ) Xplore, Google Scholar, Dimensions, and Springer. These databases cover computer science literature and provide extensive coverage of the topic and field.

### 3.4. Study Selection

A multistage screening process was conducted to select relevant articles. Initially, two reviewers screened each article based on their titles and abstract. The resulting articles were then combined into a single list, and a second full-text review was performed to make a final decision on inclusion. In any disagreement between the two reviewers, a discussion was held to reach a consensus.

### 3.5. Data Charting and Synthesis of Results

The data that were extracted from the studies meeting the inclusion criteria allowed the answering of the question listed in the Introduction: (1) the problems have been addressed involving sensor fusion techniques in animal monitoring, (2) the species have been the subject of studies, (3) the applications of sensor fusion systems for animal monitoring, (4) the sensing, processing, and interaction technologies using for animal monitoring using sensor fusion, (5) the modalities used in animal monitoring using sensor fusion, (6) how fusion techniques have been employed for analyzing animal behavior, (7) what are the reported efficacy of sensor fusion performance metrics and results obtained. The authors developed, calibrated, and utilized several templates, each containing different sections used to extract and summarize the data.

- *Related to the study.*
  - Year of publication (2011–2022).
  - Qualsyst score.
- *Related to animal monitoring.*
  - Problem addressed (e.g., increasing production, monitoring, and welfare).
  - Target species.
  - Sensor fusion target.
  - Animal activities.
  - Animal postures.
  - Number of animals included in the experiment.
- *Related to monitoring technology.*
  - Animal-computer interface (e.g., collars, vest, ear tags, and girth straps).
  - Sensor technology (e.g., accelerometer, gyroscope, magnetometer, optic flow sensor, Global Positioning System (GPS), and microphone).
  - The sensor sampling rate.
- *Related to sensor fusion*.
  - Sensor fusion type (e.g., single fusion, uni-modal switching, and mixing). Sensor fusion can be performed by fusing raw data from different sources, extrapolated features, or even decisions made by single nodes.
  - Data alignment techniques (e.g., re-sampling interpolation and timestamps). Machine learning assumes data regularity for identifying target patterns, such as fixed duration, to detect a bark in audio. Discrepancies in time, frame rates, or sensor variations (audio, video) can disrupt this regularity. Data alignment techniques are needed to establish consistent time steps in the dataset.
  - Feature extraction techniques. These techniques condense valuable information in raw data using mathematical models. Often, these algorithms are employed to reduce extracted features and optimize dataset size. A trend is using pre-trained machine learning models for feature extraction and reduction, leveraging knowledge from large-scale datasets for improved efficiency.
  - Feature type. Commonly, feature extraction techniques produce a compact and interpretative resulting dataset by applying mathematical domain transformations to the raw data. The resulting dataset can be assigned a type according to the domain, for example, time, frequency, or timefrequency domain. Raw data is collected in time series in real time. In the time domain, some representative types are traditional descriptive statistics such as mean, variance, skewness, etc. Information coming from the frequency domain can be recovered by using the Fast Fourier Transformation (FFT) or Power Spectrum analysis. Regarding time-frequency features, the short-time Fourier Transform (STFT) is the most straightforward method, but Wavelets are also employed.
  - Machine Learning (ML) algorithms. According to Goodfellow et al. [25], ML is essentially a form of applied statistics with an increased emphasis on the use of computers to statistically estimate complicated functions and a decreased emphasis on proving confidence intervals around these functions; typical classification of such methods is on supervised or unsupervised learning depending on the presence or absence of a labeled dataset, respectively. Examples of relevant ML algorithms are [26]: Gradient Descent, Logistic Regression, Support Vector Machine (SVM), K-Nearest Neighbor (KNN), Artificial Neural Networks (ANNs), Decision Tree, Back Propagation Algorithm, Bayesian Learning, and Naïve Bayes.
- *Related to performance metrics and results*.
  - Assessment techniques used to measure model performance (e.g., accuracy, f-score, recall, and sensibility) metrics used in the model evaluation should be aligned with the specific task, such as classification, regression, translation, or anomaly detection. For instance, accuracy is commonly employed in classification tasks to gauge the proportion of correct model outputs. Performance metrics are valuable for assessing model effectiveness during experiments. However, it is essential to note that model performance in real scenarios can be inconsistent due to dependencies on the available training data. This review recorded the best-reported performance metric from each article in the corresponding table.

## 4. Results

### 4.1. Overview

**Figure 2:**
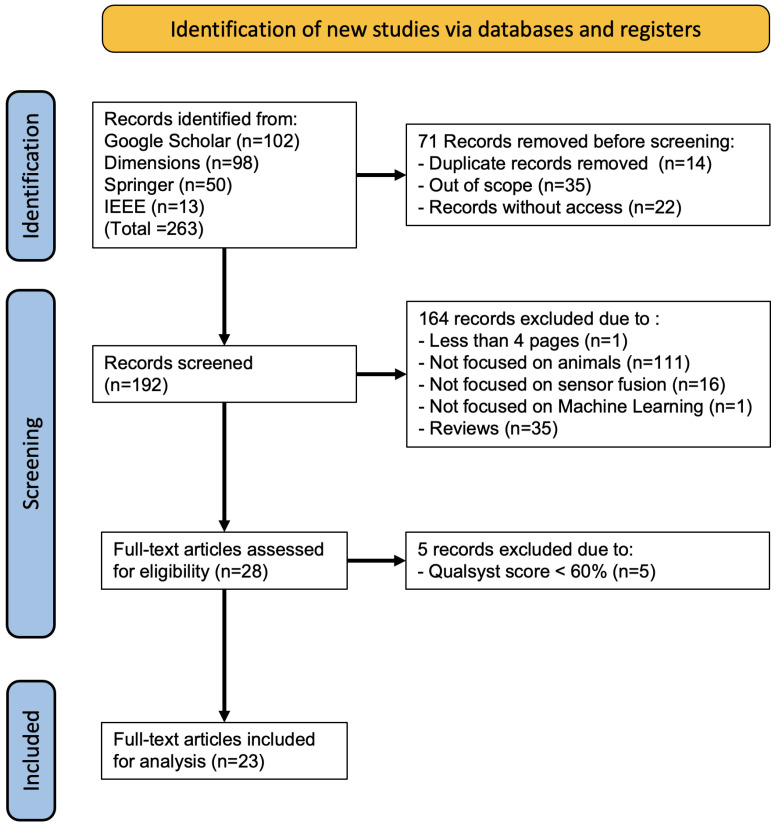
Phases of the review according to the PRISMA-ScR methodology.

During the search phase, the results showed 263 papers; after removing duplicates and pieces without access, 192 articles were obtained. Only 28 met the inclusion criteria explained earlier. The remaining 164 articles were excluded due to the following reasons: (i) have purposes not oriented to animals, (ii) use techniques not related to machine learning, (iii) use techniques not related to sensor fusion, (iv) reviews (e.g., surveys and systematic reviews), (v) articles could not be accessed, and (vi) less than four pages long. This review includes articles with Qualsyst score percentages ≥60%. As a result, 23 articles were included for in-depth analysis [7,13,14,15,16,27,28,29,30,31,32,33,34,35,36,37,38,39,40,41,42,43,44]; the remaining 5 articles were excluded since their score was below the acceptance percentage [45,46,47,48,49]. The flow diagram of the review phases’ is shown in Figure 2. The first study identified dates back to 2016 [39], and 95.7% (22/23) of the studies were conducted in the last five years (2018–2022). Most of the 23 studies were conducted in Europe (11/23, 47.8%) [7,15,16,29,30,32,33,38,39,40,41] followed by Asia (8/23, 34.7%) [13,27,34,35,36,42,43,44], and Oceania (3/23, 13%) [31,37,44]. An overview of the results is presented in Table 2, and a detailed analysis of the selected studies are displayed in Table 3, Table 4 and Table 5, according to sensor fusion level. The following subsections provide detailed findings to answer the six defined questions.

### 4.2. Problems Addressed and Target Species

**Figure 3:**
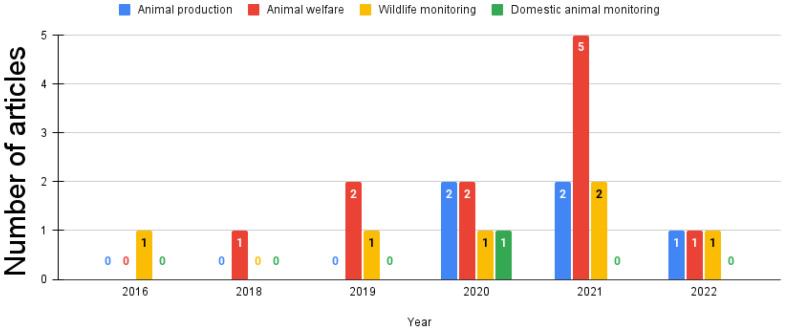
Problems addressed per year.

The reviewed papers predominantly centered around the crucial issue of animal welfare, with more than half (12/23, 52.2%) of them dedicated to this significant topic. Another critical concern explored in these papers was the imperative for the effective monitoring of animals in their natural habitats (6/23, 26.1%). Additionally, 21.7% (5/23) of the reviewed papers addressed the challenge of increasing animal production. Finally, one study (1/23, 4.3%) focused explicitly on monitoring domestic animals. Figure 3 provides an overview of the distribution of problem areas addressed per year. Regarding the targeted species, the majority of the studies (8/23, 34.8%) focused on cows. Three studies (3/23, 13%) specifically targeted horses, while there were two studies (2/23, 8.7%) for each of the following species: pigs, felines, dogs, and sheep. Finally, there was one study (1/23, 4.3%) for each one of the following species: fish, camouflaged animals, primates, koalas, goats, and birds.

### 4.3. Sensor Fusion Application

**Figure 4:**
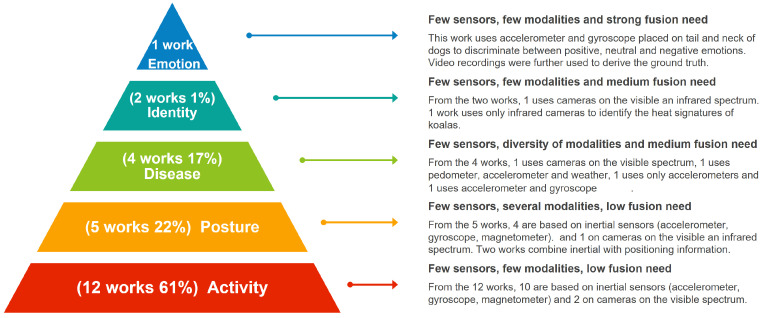
Application of technologies and sensors used.

Animal behavior monitoring using sensor fusion has been applied to various applications that can be grouped into five categories: activity detection (e.g., walking, jumping, and eating), posture detection (e.g., standing, sitting, and lying down), disease detection (e.g., hypoxia, hypothermia, and lameness), individual identification, and recognition of positive and negative emotions. As shown in Figure 4, most articles included in this review performed activity detection, and 22% performed posture detection. It is important to note that three of the articles that did activity detection also did posture detection. Fewer articles addressed the problem of detecting diseases 17%. Two articles tried to identify specific individuals, and only one worked on emotion recognition. The number of works oriented to each of the five applications seems to have a direct relationship with the complexity of the modeled behavior. That is, activity detection is a more easily detectable behavior than posture, which in turn is more easily detectable than diseases, and so on. The most challenging behavior to estimate in animals is emotion. Regarding the use of sensors in different applications, we can see that accelerometers and gyroscopes were used for all applications except for detecting individuals. Likewise, it can be seen that the information modality, the number of sensors, and the level of information fusion were related to the complexity of the behavior to be modeled. In other words, less complex behaviors, such as activities and postures, are detectable using inertial sensors or cameras without really needing a fusion of different modalities. On the other hand, identifying diseases and emotions requires that the animal carry a more significant number of sensors, use more than one information modality and merge the modalities.

### 4.4. Sensing Technology for Animal Monitoring Using Sensor Fusion

Regarding animal computer interfaces, the most used device for collecting data was the collar, which was present in 47.8% of the works, followed by the usage of natural interfaces based on computer vision in 30.4%. When it comes to sensors, they can be categorized into four main classes: motion, position, image, and others. The motion modality focused on sensors designed to measure an animal’s movements. Examples of commonly used motion sensors included accelerometers (60.86%), gyroscopes (30.43%), magnetometers (21.73%), and pedometers (4.34%). The position modality pertains to obtaining an animal’s position within its environment. Various technologies were employed for this purpose, including global positioning systems (GPS) (13.04%), wireless ad hoc systems (4.34%), global navigation satellite systems (4.34%), and real-time systems (4.34%). The optic modality involved systems that acquire images using cameras or optic flow sensors (4.34%). In this modality, the two primary camera types were visible spectrum images (34.78%) and infrared spectrum images (13.04%). Visible spectrum images capture the light visible to the human eye, while infrared spectrum images detect the heat emitted by animals. Additionally, other modalities have been discovered, such as microphone-based systems (4.34%) and weather sensors (4.34%). These modalities expand the range of sensor options available to capture data and information in different contexts.

### 4.5. Sensor Fusion Techniques

During the present review, the reviewed articles reported the usage of different sensor fusion approaches. The majority of the approaches were based on a single fusion algorithm (6/23, 26%). Other articles utilized two or more classifiers for sensor fusion (6/23, 26%). Among the articles, a subset of them (2/23, 8.6%) employed multimodal switching as a method for fusion, while another subset (2/23, 8.6%) utilized mixing. Additionally, specific fusion methods were identified, including Cross-modality interaction [36], Dual-Stream Convolutional Neural Networks [43], Path aggregation network [35], Cascade and Feedback Fusion [34], KNN-RF Weighted Fusion Model [42], and EKF [44]. Each method was mentioned once, representing 4.35% of the total.

### 4.6. Machine Learning Algorithms Used for Sensor Fusion

**Figure 5:**
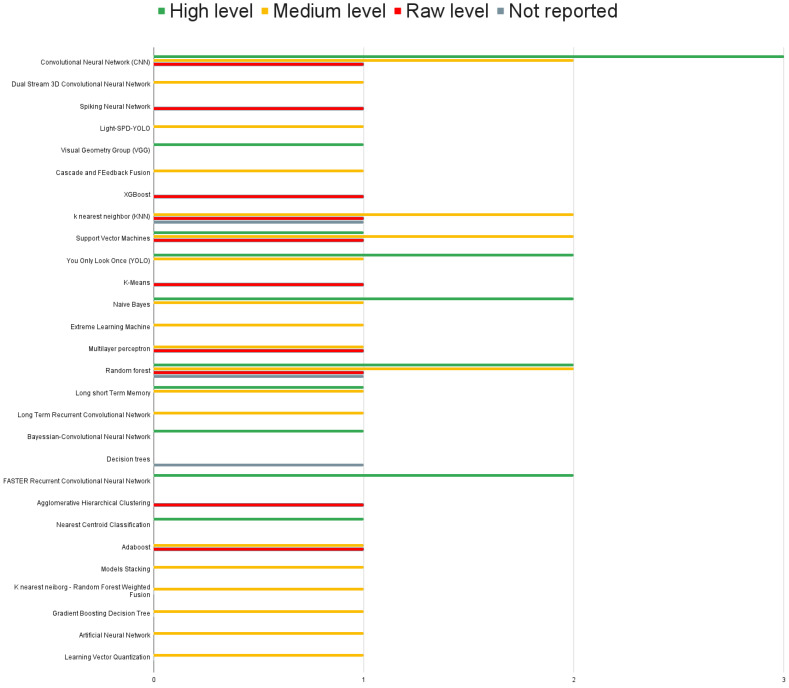
Machine learning algorithms by fusion level. Low level uses information from multiple sources without any prior data processing. At Medium level the processed data are reduced in dimensionality before being decided by an ML model. High level applies decision algorithms to ML output.

In the reviewed articles, an analysis was performed to determine the prevailing trends in selecting learning algorithms for behavior detection in animals. However, no clear consensus emerged regarding the preference for specific types of algorithms. Figure 5 shows no dominant machine-learning algorithm was utilized in sensor fusion to recognize animal behavior. In particular, the two most frequently employed techniques were random forest and convolutional neural networks (CNNs). Both algorithms found application in all three categories of sensor fusion. Support vector machines (SVM) and K-Nearest Neighbors (KNN) were employed in multiple reviewed studies. It should be noted that these four algorithms share a common attribute, namely their status as standard approaches that have demonstrated favorable outcomes across diverse data types and applications. Often regarded as baseline algorithms, they serve as a foundation upon which more specialized algorithms can be developed to address specific problems in the classification domain. This observation prompts consideration of the current state of research and development in sensor fusion algorithms for multisensor and multimodal recognition of animal behavior. It becomes apparent that this field is still in its nascent stages, as indicated by the continued reliance on these foundational techniques. Pursuing more advanced and tailored algorithms represents an opportunity for further exploration, potentially leading to improved accuracy and performance in animal behavior recognition.

### 4.7. Performance Metrics and Reported Results

This scoping review provides an extensive overview of the performance metrics commonly utilized in studies related to sensor fusion. Sensor fusion plays a crucial role in integrating data from multiple sensors to enhance the reliability of systems. Throughout our analysis, we identified several prominent metrics, including accuracy [7,13,15,16,27,29,32,33,35,36,38,39,40,41,43,50,51], recall [15,27,36,39,41,43], and F1-score [15,27,29,36,37,41], which are widely recognized and essential for evaluating fusion system performance. The accuracy metric serves as a fundamental measure in sensor fusion studies, assessing the overall correctness of the fused data. It quantifies the proportion of instances or predictions that are accurately classified in relation to the total number of instances. Notably, a significant portion of the included articles (78%) incorporated accuracy into their evaluation. Among the studies reporting accuracy results, the average accuracy was determined to be 92.91% across the various levels of fusion. Additionally, recall, also known as sensitivity, emerged as another critical metric employed by the sensor fusion proposals in this review. Recall measures the system’s ability to correctly identify true positive cases, providing insights into its performance in capturing all relevant positive instances. Among the studies that reported their results using this metric (34.78%), the average recall was found to be 87.54%. Furthermore, the F1-score proved to be a vital metric that combines precision and recall, offering a comprehensive evaluation of sensor fusion system performance. By considering both the system’s ability to identify positive instances (precision) correctly and its capability to capture all relevant positive instances (recall), the F1-score provides a balanced assessment. Among the studies that reported their results using this metric (21.73%), the average F1-score was determined to be 82.04%. While accuracy, recall, and F1-score were commonly employed metrics in the reviewed sensor fusion studies, it is important to note that other metrics were also identified. These additional metrics include the Receiver Operating Characteristic curve (ROC), Matthews correlation coefficient (MCC), Silhouette analysis, Kappa Value, Specificity, Recognition Error Rate, Mean Average Precision, Recognition Rate, Youdens Index, and Cohens. These metrics offer valuable insights into different aspects of fusion system performance, showcasing the diverse approaches used in evaluating sensor fusion systems.

### 4.8. Analysis by Sensor Fusion Level

**Figure 6:**
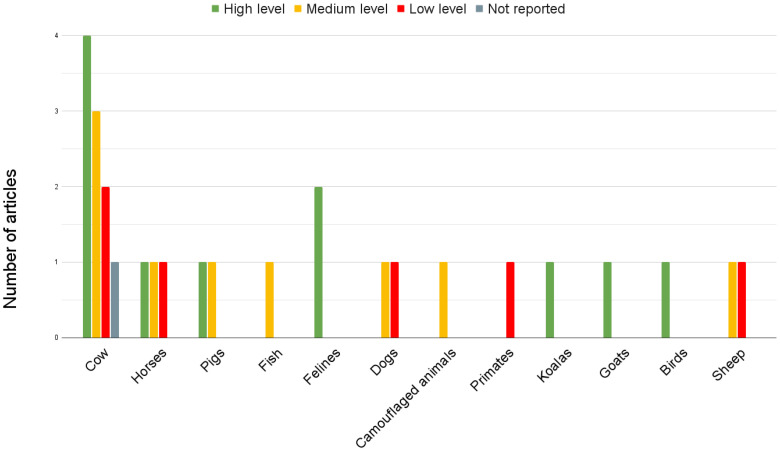
Target species presented by fusion level. Low level uses information from multiple sources without any prior data processing. At Medium level, the processed data are reduced in dimensionality before being decided by an ML model. High level applies decision algorithms to ML output.

**Figure 7:**
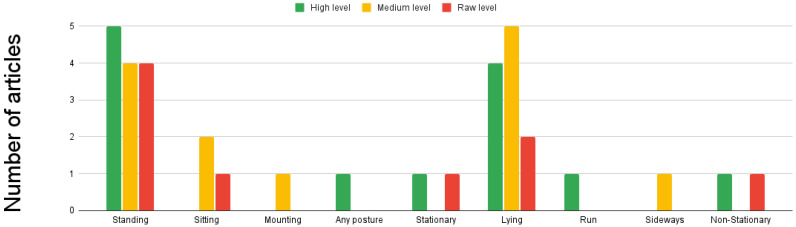
Posture detection by fusion level. Low level uses information from multiple sources without any prior data processing. At Medium level, the processed data are reduced in dimensionality before being decided by an ML model. High level applies decision algorithms to ML output.

**Figure 8:**
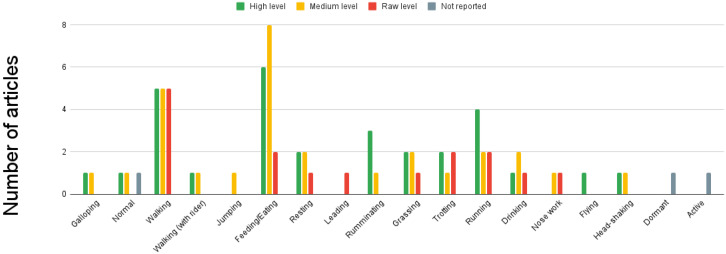
Activity detection by fusion level. Low level uses information from multiple sources without any prior data processing. At Medium level the processed data are reduced in dimensionality before being decided by an ML model. High level applies decision algorithms to ML output.

**Figure 9:**
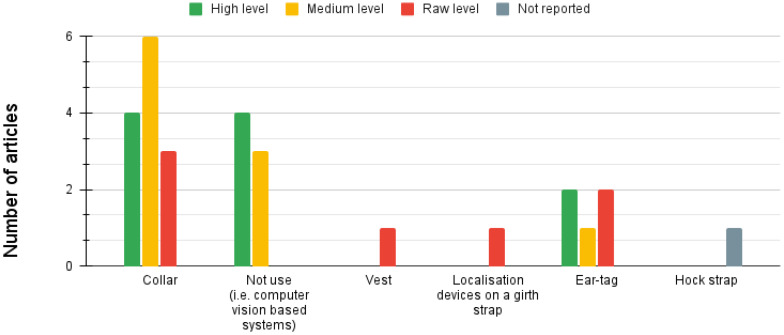
Animal computer interfaces by fusion level. Low level uses information from multiple sources without any prior data processing. At Medium level the processed data are reduced in dimensionality before being decided by an ML model. High level applies decision algorithms to ML output.

**Figure 10:**
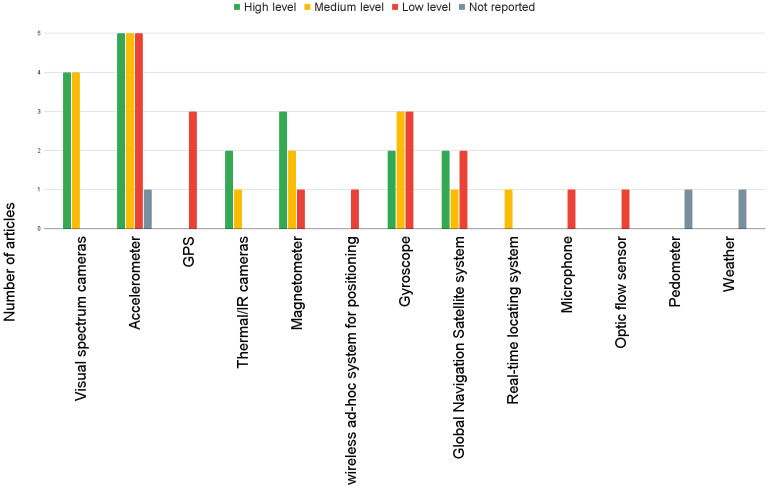
Sensors by fusion level. Low level uses information from multiple sources without any prior data processing. At Medium level the processed data are reduced in dimensionality before being decided by an ML model. High level applies decision algorithms to ML output.

Among the included articles, we observed the following distribution: the low level of sensor fusion was employed in 30.4% of the papers. In comparison, the feature level of fusion was utilized in 39.1% of the included papers. Additionally, a high level of sensor fusion was present in 34.7% of the papers. It is important to note that for more detailed information on each level of fusion, readers can refer to Table 3, Table 4, and Table 5, respectively, which provide comprehensive data on the specific techniques and approaches employed in each level. It should be emphasized that we came across a paper where the level of fusion employed in their approach was not reported. (reference: [30]). Sensor fusion is a versatile technique that can be applied to various animal populations. In particular, cows [28,30,37,38,40,41,42,44] and horses [29,36,39] are among the animal populations that are covered by all three levels of sensor fusion. However, sensor fusion can be applied to other animal populations. In the high level: pigs [33], felines [13,32], koalas [31], goats [29], and birds [32], regarding feature level: pigs [35], fish [43], dogs [27], camouflaged animals [34], and sheep [14]. Meanwhile, for the low-level: horses [39], dogs [7], primates [16], and sheep [15]. For further details, Figure 6 can be consulted. The predominant sensor fusion approach is feature-level, which covers 6 of the 9 applications identified in the study, which were: detection of activity [14,27,36,43], poses [35,36], camouflaged animals [34], emotions, spatial proximity [38], and [27], social behavior analysis [38]. Regarding animal posture detection, standing and lying postures are covered by all three levels of sensor fusion. However, any moving posture [32] and running [32] poses are only covered by the high level of sensor fusion. The feature-level sensor fusion only detects mounting [35] and sideways [27] postures. Notably, the low level of sensor fusion does not cover any specific posture not covered by the higher levels. Consult Figure 7 for additional information. The three levels of sensor fusion can detect a range of animal activities, including walking, feeding, resting, grazing, trotting, running, and drinking. However, flying [32] activity is only detected using a high level of fusion, while jumping [27] is only approached using feature fusion. Meanwhile, leading is only detected using low fusion. See Figure 8 for more details. The study found that wearable animal-computer interfaces were used primarily at a low fusion level. Wearable devices, such as collars, vests, girth straps, and ear tags, can aid in collecting data from different sensor types. This aligns with research that focuses on natural interfaces, such as computer vision systems, which did not include proposals that utilized low-level fusion. For further information, see Figure 9. Regarding the sensors utilized in the analyzed papers, it was observed that accelerometers, gyroscopes, and magnetometers were consistently employed across all three levels. These sensors are very relevant to the primary applications of activity and pose detection. Additionally, camera usage was reported at high and feature levels, with both visual spectrum and IR cameras being used. Notably, the proposals at all three levels of sensor fusion relied on the same set of sensors, which were mainly related to movement; this is illustrated in Figure 10.

## 5. Discussion

### 5.1. Overview

Given the increasing interest in machine learning-based sensor fusion techniques for animal monitoring, future studies in this field must identify key features that designers of this type of system should consider. This scoping review contributes by identifying and summarizing essential characteristics that currently define the construction and evaluation of these systems. The findings of this review offer valuable insights for future research in this area and contribute to the advancement of animal science. Our study examined 23 serious games published over the past 12 years (2011–2022). Notably, the majority of these publications (22 out of 23, 95.7%) were released in the last five years, starting in 2018. The earliest study we identified dates back to 2016, while 2021 had the highest number of publications, with nine articles. Furthermore, the articles reviewed demonstrated an average Qualsyst score of 81.1 ± 14.02%, indicating a favorable level of quality and robustness.

### 5.2. Problems Addressed and Target Species

Regarding the problems addressed, most of the studies primarily focused on animal welfare, followed by wildlife animal monitoring and increasing production (see Figure 3). Notably, there has been an observable upward trend in animal welfare studies from 2018 to 2021, indicating a growing emphasis on improving animal welfare in recent years. These tendencies align with the advancements witnessed in sensor-based animal monitoring, which have revolutionized our understanding of and interaction with animals. Specifically, sensor-based animal monitoring has substantially contributed to three key areas: animal welfare [52], wildlife conservation [53], and livestock management [54]. Animal welfare encompasses the ethical and compassionate treatment of animals, taking into account their physical, mental, and emotional well-being. Wildlife conservation entails protecting, preserving, and managing wild animal species and their habitats. Livestock management involves the raising and caring of domesticated animals, primarily for agricultural purposes such as food and fiber production or labor. Interestingly, there was a notable lack of work focusing on monitoring domestic animals, such as dogs, presenting an opportunity for further study.

In terms of the target species, the majority of the studies (16/23, 69.56%) focused on farm animals, with cows being the most commonly studied species (8/23, 34.8%), followed by horses (2/23, 8.7%), pigs (2/23, 8.7%), sheep (2/23, 8.7%), goats (1/23, 4.3%), and fish (1/23, 4.3%). Additionally, six studies (26.1%) focused on wild animals, including wild horses, felines, camouflaged animals, primates, koalas, and birds. Surprisingly, only two papers specifically focused on domestic animals, such as dogs (8.7%). The predominance of farm animal studies can be attributed to their significant commercial, nutritional, and health importance [54]. In contrast, the focus on wild animals reflects the growing interest in wildlife conservation and management [53]. However, despite their global population and societal relevance, there were a limited number of studies addressing domestic animals, particularly dogs and cats. Further research in this area is warranted to handle these animals’ monitoring and welfare needs. It is essential to highlight the considerable variation in the number of participants involved in the data collection process across species. As expected, studies involving farm animals typically included a large number of participants, such as 697 cows or 2404 pigs. Conversely, studies involving wild animals had fewer participants, for instance, three wild horses, 48 koalas, or 26 primates. Interestingly, the number of participants in studies focusing on domestic animals was unexpectedly lower than those for wild animals, with only nine or ten dogs, for example. Further research and standardized approaches are necessary to ensure adequate sample sizes and representative data collection across different animal species.

### 5.3. Sensor Fusion Application

The majority of the articles included in the review focused on activity recognition (52%), posture recognition (47.8%), and health detection (17.3%). However, several other interesting research areas could be explored within these domains. In terms of activity detection, while walking (65.21%), feeding or eating (60.8%), and running (30.4%) received significant attention, it could also be valuable to investigate other activities such as sleeping or animals’ vocalizations, such as barking in dogs.

Regarding posture detection, the included papers primarily focused on standing (52%) followed by laying, including postures such as lying on the belly and on the side (44.4%). However, additional postures, such as the "play bow" posture in dogs, could be explored, a common invitation to play.

Regarding health systems, the predominant focus was lameness detection (8%), but there are other potential applications worth exploring, including hypoxia, hypothermia, ketosis, and ischemia detection. Similarly, emotion detection was relatively limited in the reviewed articles, with only one paper evaluating positive, neutral, and negative emotions in animals. It could be interesting to incorporate biometric sensors that provide insightful information about an animal’s health condition and emotions, such as surface skin temperature, heart rate, and respiratory rate, to name a few.

Overall, while the reviewed articles covered important areas such as activity recognition, posture detection, and health monitoring, there is still room for exploration and expansion into other activities, postures, health indicators, and emotional aspects in animal sensor fusion applications.

### 5.4. Sensing Technology

For data collecting, our investigation revealed that the most used device was the collar (47.82%) and ear tags (17.39%). On the other hand, a significant number of proposals used computer vision-based systems. Therefore, they did not use a wearable interface (30.43%). Regarding sensors, the accelerometer was the primarily used device (60.86%) which was present in 14 of 23 articles, followed by visible spectrum cameras (34.78%), magnetometers (21.73%), gyroscopes (30.43%). As these types of sensors can commonly be found in devices such as smartphones, it might be possible to utilize them during the prototyping or validation stages. However, their high cost and logistical implications could hinder their use in large-scale animal applications, such as monitoring cows or other farm animals. Similarly, developing alternatives to collars and ear tags for animal computer interfaces (i.e., vests, and wristbands) can be challenging due to the anatomical features of each animal species. Each species may have specific physical characteristics that need to be considered when designing and implementing these alternative interfaces.

One notable observation from the reviewed articles is the limited utilization of biometric sensors. These specialized sensors, capable of measuring vital parameters such as heart rate, respiration rate, body temperature, and blood pressure, offer invaluable insights into an animal’s health, emotions, and complex behaviors. However, the adoption of such sensing technology is hindered by the scarcity of animal-specific sensors in the market. Moreover, although some commercial sensors are already available that can measure biometric parameters such as heart rate, respiratory rate, and body temperature, they tend to be costly, have limited distribution, and do not always meet the researchers’ expectations.

In the coming years, the rapid advancement of IoT technology is expected to profoundly impact animal monitoring systems in various settings such as farms, stables, and homes. The IoT offers immense potential for scaling up animal monitoring systems, as its infrastructure enables the integration of hundreds or even thousands of sensors distributed across different locations. In IoT applications, three distinct levels of data processing can be identified: edge computing, fog computing, and cloud computing. These different processing paradigms offer the flexibility to implement robust sensor fusion systems that leverage machine learning techniques based on the specific context.

At the edge computing level, data processing occurs directly within the data sensing devices themselves. This decentralized approach allows for real-time processing and decision-making, making it particularly suitable for scenarios where internet access may be limited or unreliable, such as wildlife monitoring. Fog computing, on the other hand, leverages the computing and storage resources available in devices and systems located closer to users and data sources. This intermediate level of processing enables efficient data aggregation and local analysis. Lastly, cloud computing involves storing and processing data in a remote infrastructure connected to the data source through the Internet. This level of processing is well-suited for applications in environments with reliable connectivity, such as farms or homes, where a wider range of connectivity options is available.

### 5.5. Levels of Sensor Fusion

The review indicates that sensor fusion has been applied to different animals, with a particular focus on cows and horses. However, there were also studies involving pigs, felines, koalas, goats, birds, fish, dogs, camouflaged animals, and sheep, among others. This highlights the versatility of sensor fusion across different animal species.

The reviewed articles primarily focused on activity and pose detection as the main applications of sensor fusion. These categories were addressed across all three levels of fusion, highlighting their importance in understanding animal behavior. Our findings also revealed a study that specifically aimed to evaluate personality using a low level of fusion. Feature level fusion, on the other hand, was utilized for various applications such as social behavior analysis, camouflaged animal detection, emotion detection, and spatial proximity evaluation. These applications required the integration of data from multiple sensors to analyze and interpret complex behavioral patterns. In terms of individual recognition, a high level of sensor fusion was employed. This fusion level allowed for accurate identification and tracking of individual animals within a population. Furthermore, health detection was covered by both the low and high levels of sensor fusion. This indicates that sensor fusion techniques could be used to monitor and detect various health parameters and conditions in animals. Using low-level sensor fusion in challenging applications such as personality evaluation and health detection can be counter-intuitive and may stem from constraints during the system development stage. This can be attributed to the reliance on machine learning-based sensor fusion, which heavily depends on the availability and quality of data.

Regarding the use of sensors across the three levels of fusion, it was observed that systems based on natural interfaces such as cameras were primarily utilized in the high or feature levels. This is because digital images captured by cameras require processing to extract useful information. In contrast, the raw level of fusion focuses on sensor data that does not require extensive processing, making it less suitable for incorporating camera-based systems. The usage of accelerometers, gyroscopes, and magnetometers was widely employed across all three levels of fusion. The reason for this could be that activity and pose detection systems primarily rely on movement data. These sensors are specifically designed to capture motion-related information, making them suitable for those applications. Interestingly, activity recognition and posture detection were identified as two of the most popular research applications in this review. Another intriguing finding was that the low level of fusion presented the most sensor variety, incorporating sensors such as positioning systems, microphones, and optic flow sensors that were not utilized in the higher levels. This suggests that the low level of sensor fusion allows researchers to incorporate a broader range of sensor inputs without significantly increasing resource requirements. Similarly, when considering animal-computer interfaces, it can be observed that the low level of fusion offers a wider variety of options than the higher levels. At the low level, researchers have the flexibility to employ various interface devices such as collars, vests, girth straps, and ear tags.

The use of different sensor fusion levels can present difficulties and limitations. This review was presented in a three-level categorization. One advantage of utilizing low-level sensor fusion is its ability to work with a relatively small amount of data and operate on systems with lower computational requirements. Nevertheless, this comes with a possible tradeoff with accuracy, precision, or other important performance metrics. The fusion of raw sensor data may result in less refined and less informative output. The other extreme of sensor fusion is high-level fusion. This type of sensor fusion typically requires at least two trained models. However, high-level fusion also comes with its own set of challenges. One significant challenge is requiring a substantial amount of representative data to train and fine-tune the individual models involved in the fusion process. Acquiring and labeling such data can be time-consuming and resource-intensive. Moreover, the computational power needed to run multiple trained models simultaneously can be demanding. High-level fusion often involves complex computations and may require significant computational resources, limiting its practicality in resource-constrained environments or real-time applications. Furthermore, the performance and accuracy of the trained models heavily rely on the quality and representativeness of the training data. Insufficient or biased data can negatively impact the effectiveness of the high-level approach. In between low-level and high-level fusion is the medium level, which, as its name suggests, offers a balance between the two extremes. Medium-level or feature fusion combines sensor data at an intermediate level of abstraction. This approach aims to leverage the advantages of low-level and high-level fusion while mitigating some limitations. Medium-level fusion can provide a more meaningful representation of the sensor data than low-level fusion, capturing relevant features and reducing noise or redundancy. At the same time, it can offer a more computationally efficient and less data-intensive alternative to high-level fusion. The selection of the appropriate fusion level depends on factors such as the complexity of the application, the available resources, and the desired trade-off between accuracy and computational requirements.

### 5.6. Sensor Fusion Techniques

The usage of different sensor fusion approaches was reported in the reviewed articles, reflecting the diversity in fusion techniques. The majority of the methods were based on a single fusion algorithm, indicating the prevalence of this approach in the field. Researchers often rely on a specific fusion algorithm that best suits their application requirements. Moreover, the incorporation of two or more classifiers for sensor fusion indicates an acknowledgment of the advantages associated with combining multiple classifiers to enhance accuracy and robustness in fusion systems. Additionally, the search conducted for this review yielded articles that proposed new sensor fusion techniques.

The increasing use and proposal of newer sensor fusion techniques in research indicate that the field of sensor fusion is experiencing significant growth. This trend suggests that researchers are actively exploring and developing innovative approaches to improve the effectiveness and efficiency of sensor fusion systems. By introducing novel fusion techniques, researchers aim to address existing limitations, enhance sensor fusion capabilities in various domains, and advance the overall state-of-the-art in this field.

### 5.7. Machine Learning Algorithms

During the conducted review, a wide range of machine learning algorithms focused on sensor fusion were identified. The diversity of these algorithms suggests that sensor fusion is a rapidly growing research area, with researchers actively exploring various techniques to address different challenges and application requirements. This indicates a strong interest in advancing the field of sensor fusion and improving its effectiveness in animal-focused applications. Our findings show that neural networks-based approaches and random forest algorithms are widely used, which could indicate their efficacy and suitability for sensor fusion tasks.

Another interesting observation is that, besides Convolutional Neural Networks, there does not seem to be a clear preference for a specific machine learning algorithm across different fusion levels. This suggests that researchers are exploring various algorithms and considering their suitability for different fusion levels based on the specific requirements of their applications. This indicates a flexible approach in selecting machine learning algorithms for sensor fusion, where the focus is more on achieving optimal results rather than relying on a specific algorithm for a particular fusion level.

### 5.8. Performance Metrics and Reported Results

Based on the results reported in the included studies, it was evident that there was no clear preference for using specific performance metrics in sensor fusion. The choice of metrics appears to depend more on the algorithms employed and the specific objectives of the research. Different algorithms may have distinct evaluation requirements, influencing the selection of appropriate metrics that align with their unique characteristics and goals. Nevertheless, it is worth noting that among the metrics used in this review, accuracy, recall, and F1-score were the most commonly employed. The average values obtained for these metrics were 92%, 87%, and 82%, respectively. These high average values suggest that the sensor fusion systems evaluated in the studies exhibited good performance.

### 5.9. Limitation of the Review

The scoping literature review has limitations that should be considered when interpreting the results. First, our inclusion criteria specifically targeted data and sensor fusion techniques using machine learning for animal monitoring, excluding studies that explored sensor use without fusion techniques and potentially limiting the breadth of our findings. Second, our search strategy was constrained by database selection, although we attempted to mitigate this limitation by searching multiple databases. In addition, we included articles with a Qualsyst score of >=60% to ensure minimal experimental criteria. However, this threshold may have excluded studies with valuable insights that did not meet the specific scoring criteria. The limited number of included studies and the possibility of missing relevant studies during our search process also impact our review’s comprehensiveness impact developing. Despite these limitations, this study provides an essential overview of the current state of sensors and data fusion techniques for animal monitoring, offering valuable insights into important characteristics, reported evaluations, and existing constraints in the field.

## 6. Conclusions

This review presents the usage of sensor fusion and machine learning focused on livestock and companion animals. This article aims to provide valuable insights to a diverse group of readers focused on animal studies. The search was conducted in IEEE Xplore, Google Scholar, Dimensions, and Springer databases using the search words “sensor fusion machine learning animals”. The articles were filtered according to the language, page length, content, publication date, and Qualsyst Score above 60%. Only 23 of 263 initially retrieved met the inclusion criteria (8.67%).

The review categorizes sensor fusion into Low/Raw, Medium/Feature, and High/ Decision. Our research indicates that there has been an increase in the topic of sensor fusion related to animals in recent years. However, the majority of studies focus only on cows, neglecting other animal populations such as dogs, sheep, and cats, to name a few examples. It is possible to believe that the main focus is on cow-related applications due to their importance in the global dietary industry. Additionally, most studies rely on collar-based animal-computer interfaces, indicating an opportunity to develop solutions based on ear tags, vests, or other types of wearable devices and also explore new proposals for Animal-Computer Interaction methods. Recent advances in artificial intelligence, machine learning, and computing power on portable devices have opened the doors to the possibility of proposing new natural interaction methods based on animal vocalizations or learning from their behavioral patterns. While the reviewed articles primarily focus on posture and activity recognition, there is a small number of articles related to emotion detection and health monitoring, indicating the potential for the development of systems addressing these topics. In terms of machine learning algorithms, there is no particular architecture for performing the fusion. However, neural networks, SVM, and random forest-based solutions were found across the three levels of sensor fusion, suggesting that these algorithms are a good starting point for developing such projects. The most commonly used sensors include accelerometers, gyroscopes, magnetometers, and visual spectrum cameras. It is surprising that none of the included articles utilized biometric sensors. The usage of sensors specialized in biological signals, such as electrocardiogram, electromyography, electroencephalography, respiratory rate, and noninvasive skin surface temperature sensors designed explicitly for animals, could lead to the creation of better products aiming at improving not only production but also animal well-being in general. This could potentially meet with design challenges, such as creating one specific sensor for just one animal species that could be commercially nonviable. Additionally, animals present different skin properties, which can limit the application of optical and electrode-based devices. 

## Figures and Tables

**Table 1:** QualSyst selected questions [24].

Question
1. Is the research question/objective sufficiently described?
2. Is the study design evident and appropriate?
3. Is the selection of subject/comparison group or source of information/input variables
described and appropriate?
4. Are the subject (and comparison group, if applicable) characteristics sufficiently described?
5. If it is an interventional study, and random allocation was possible, is it described?
6. If it is an interventional study and blinding of investigators was possible, is it reported?
7. If it is an interventional study, and blinding of subjects was possible, is it reported?
8. Are the outcome and (if applicable) exposure measure(s) well-defined and robust
to measurement/miss-classification bias? Are the means of assessment reported?
9. Are the analytic methods described/justified and appropriate?
10. Is some estimate of variance reported for the main results?
11. Was confounding controlled for?
12. Are the results reported in sufficient detail?
13. Are the conclusions supported by the results?

**Table 2:** Overview of the main characteristics of the reviewed works (N = 23).

Characteristic		Studies, n (%)
**Year of publication**		
	2022	4 (17.3%)
	2021	9 (39.1%)
	2020	5 (21.7%)
	2019	3 (13%)
	2018	1 (4.3%)
	2017	0 (0%)
	2016	1 (4.3%)
**Problems addressed**		
	Animal welfare	11 (47.8%)
	Wildlife monitoring	6 (26.1%)
	Animal production	5 (21.7%)
	Domestic animal monitoring	1 (4.3%)
**Target species**		
	Cows	8 (34.8%)
	Horses	3 (13%)
	Pigs	2 (8.7%)
	Felines	2 (8.7%)
	Dogs	2 (8.7%)
	Sheep	2 (8.7%)
	Fish	1 (4.3%)
	Camouflaged animals	1 (4.3%)
	Primates	1 (4.3%)
	Koalas	1 (4.3%)
	Goats	1 (4.3%)
	Birds	1 (4.3%)
**Sensor fusion application**		
	Activity detection	12 (52.2%)
	Posture detection	11 (47.8%)
	Health screening	4 (17.4%)
	Identity recognition	2 (8.6%)
	Camouflaged animal detection	1 (4.3%)
	Emotion classification	1 (4.3%)
	Personality assessment	1 (4.3%)
	Social behavior analysis	1 (4.3%)
	Spatial proximity measurement	1 (4.3%)
**Animal-computer interface**		
	Collar	11 (47.8%)
	Natural (vision, audio)	7 (30.4%)
	Ear-tag	4 (17.4%)
	Vest	1 (4.3%)
	Localization devices on a girth strap	1 (4.3%)
	Hock strap	1 (4.3%)
**Sensors technology**		
	Accelerometer	14 (60.7%)
	Camera (visual spectrum range)	8 (34.8%)
	Gyroscope	7 (30.4%)
	Magnetometer	5 (21.7%)
	Global Positioning System (GPS)	3 (13%)
	Camera (IR spectrum range)	3 (13%)
	Global Navigation Satellite System	2 (8.7%)
	Pedometer	1 (4.3%)
	Wireless ad-hoc system for positioning	1 (4.3%)
	Real-time locating system	1(4.3%)
	Microphone	1 (4.3%)
	Optic flow sensor	1 (4.3%)
	Weather sensor	1 (4.3%)
**Fusion level**		
	Features/medium level	9 (39.1%)
	Classification/regression/late/decision	8 (34.8%)
	Raw/Early fusion	7 (30.4%)
	Not specified	1 (4.3%)

**Table 3:** Sensors fusion for animal monitoring and assessment (Low/Raw level).

Author	Rios-Navarro et al. [39]	Kasnesis et al. [7]	Leoni et al. [16]	Xu et al. [44]	Arablouei et al. [28]	Kaler et al. [15]
**Year**	2016	2022	2020	2020	2022	2020
**Qualsyst Score**	76%	86%	100%	100%	100%	61%
**Problem addressed**	Wildlife monitoring	Domestic animal monitoring	Wildlife monitoring	Increase production	Increase production	Welfare
**Fusion application**	Activity detection	- Activity detection - Posture detection	Posture detection	- Personality assessment - Influence of individuals	Activity detection	Health detection
**Target population**	Horses	Dogs	Primates	Cows	Cows	Sheep
**Animal interface**	Collar	Vest	Collar	Girth strap	- Collar - Ear-tag	Ear-tag
**Activities**	- Walking - Trotting	- Walking - Trotting - Running - Nose work	- Walking - Running - Feeding - Eating	Leading	- Walking - Resting - Drinking - Grassing	Walking
**Postures**	Standing	Standing	- Standing - Sitting	- Stationary - Non-stationary - Closeness between individuals	-	- Standing - Lying on the belly, - Lying on the side
**Participants**	3	9	26	10	8	18
**Sensors**/ **Sampling rate (Hz, FPS)**	- Accelerometer - Magnetometer - Gyroscope - GPS	- Accelerometer (100 Hz) - Magnetometer - Gyroscope (100 Hz) - GPS (10 Hz)	- Accelerometer (12 Hz) - GPS - Optic flow sensor	- GPS (1 Hz) - Wireless ad-hoc system for positioning	- Accelerometer (50 Hz, 62.5 Hz) - GPS - Optic flow sensor	- Accelerometer (16 Hz) - Gyroscope
**Feature extraction**	-	ML models (LSTM-models, CNN-models, etc.)	- Acceleration norm - Acceleration ratios	- ML models (LSTM-models, CNN-models, etc.)	-	-
**Feature type**	-	Spatiotemporal	- Spatiotemporal - Motion	- Spatiotemporal - Spatial - Closeness	- Spatiotemporal - Spatial	Motion Statistical
**Data alignment**	-	Same datasize	Downsampling	Timestamps	Timestamps	Timestamps
**Machine learning algorithms**	Spiking Neural Network	Convolutional Neural Networks	XGBoost	- K-Means - Agglomerative Hierarchical Clustering	Multilayer perceptron	- KNN - Random forest - SVM - Adaboost
**Sensor fusion type**	Single fusion algorithm	Single fusion algorithm	Single fusion algorithm	Extended Kalman filter (EKF)	- Multimodal switching - Two or more classifiers	Mixing
**Performance metrics**	Accuracy (83.33%)	Accuracy (93%)	- Accuracy (100%) - Sensibility/Recall (100%) - Specificity (100%)	-Accuracy (15 cm), - Silhouette analysis	Matthews correlation coefficient	-Accuracy (91.67%) - Sensibility/Recall (≈80%) - Specificity (≈70%) - Precision (≈75%) - F1-score (≈85%)

**Table 4:** Sensors fusion for animal monitoring and assessment (Features/medium level).

Author	Ren et al. [38]	Mao et al. [36]	Wang et al. [43]	Luo et al. [35]	Huang et al. [34]	Aich et al. [27]	Jin et al. [14]	Tian et al. [42]	Arablouei et al. [28]
**Year**	2021	2021	2021	2021	2021	2019	2022	2021	2022
**Qualsyst Score**	69%	100%	69%	69%	64%	78%	100%	65%	100%
**Problem addressed**	Increase production	Welfare	Welfare	Welfare	Wildlife monitoring	Welfare	Welfare	Increase production	Increase production
**Fusion application**	Social behavior analysis	- Posture detection - Activity detection	- Activity detection - Health detection	Posture detection	camouflaged animals detection	- Activity detection - Emotion detection	Activity detection	- Activity detection - Posture detection	Activity detection
**Target population**	Cows	Horses	Fishes	Pigs	Camouflaged animals	Dogs	Sheep	Cows	Cows
**Animal interface**	Collar	Collar	-	-	-	Collar	Collar	Collar	- Collar - Ear-tag
**Activities**	-	- Galloping - Trotting - Feeding/eating - Walking	- Normal - Feeding/eating	-	-	- Normal - Feeding/eating - Nose work - jumping	- Walking - Running - Grassing	- Walking - Running - Feeding - Eating - Resting - Drinking - Head-shaking - Ruminating	- Walking - Resting - Drinking - Grassing
**Postures**	-	Standing	-	- Standing, - Lying on belly - Lying on side - Sitting - Mounting	-	- Sitting - Sideways - Stay	- Standing - Lying on belly - Lying on the side - Sitting - Standing	-	-
**Participants**	120	18	-	2404	-	10	3	60	8
**Sensors**/ **Sampling rate (Hz, FPS)**	- Visual spectrum camera (25 FPS) - Real-time locating system	- Accelerometer (100 Hz) - Magnetometer - Gyroscope (12 Hz)	Visual spectrum camera (25 FPS)	- Visual spectrum camera (15 FPS) - Infrared thermal camera	Visual spectrum camera	- Accelerometer (33 Hz) - Gyroscope	- Accelerometer (20 Hz) - Gyroscope	- Accelerometer (12.5 Hz) - Magnetometer	- Accelerometer (50 Hz, 62.5 Hz) - GPS - Optic flow sensor
**Feature extraction**	ML models (LSTM-models, CNN-models, etc.)	ML models (LSTM-models, CNN-models, etc.)	ML models (LSTM-models, CNN-models, etc.)	ML models (LSTM-models, CNN-models, etc.)	ML models (LSTM-models, CNN-models, etc.)	-	-	Randomly selected features	-
**Feature type**	Temporal domain	- Spatiotemporal - Temporal domain	- Spatiotemporal - Motion	Spatial	Spatial	- Statistical - Peak-based features	- Frequency domain - Statistical	- Temporal domain	- Spatiotemporal - Spatial
**Data alignment**	- Timestamps - Mapping	Concatenation	- Concatenacton - Downsampling - Same datasize	- Concatenation - downsampling - same datasize	Same datasize	Timestamps	Same datasize	-	Timestamps
**Machine learning algorithms**	- Convolutional Neural Networks - Long Short-Term Memory - Long-Term Recurrent Convolution Networks	Convolutional Neural Networks	DSC3D network	- Light-SPD-YOLO model - YOLO	- Cascade - Feedback Fusion	- SVM - KNN - Naive Bayes - Random forest - Artificial Neural Networks	ELM Adaboost Stacking	- SVM - KNN - Random forest - KNN-RF Weighted Fusion - Gradient Boosting Decision Tree - Learning Vector Quantization	Multilayer perceptron
**Sensor fusion type**	Single fusion algorithm	Cross-modality interaction	Dual-stream Convolutional Neural Networks	Path aggregation network	- Cascade - Feedback Fusion	Single fusion algorithm	Two or more classifiers	KNN-RF weighted fusion model	Multimodal switching of two or more classifiers
**Performance metrics**	- Accuracy (93.2%) - Confusion matrix (92%)	- Accuracy (93.3%) - Sensibility/Recall (83.7%) - F1-score (82.9%)	- Accuracy (95.7%) - Sensibility/Recall (100%) - F1-score - Confusion matrix	- Sensibility/ Recall - Accuracy (98.4%) - Receiver operating characteristic curve - Mean average precision (97.7%)	- Accuracy	- Accuracy (96.5%) - Sensibility/Recall (94.6%) - F1-score (93.6%) - Confusion matrix	- Accuracy (99.7%) - Kappa value (0.995)	-Accuracy (99.3%) - Confusion matrix - Recognition error rate - Recognition rate (98.5%)	Matthews correlation coefficient

**Table 5:** Sensor fusion for animal monitoring and assessment (Decision/High level).

Author	Hou et al. [33]	Feng et al. [13]	Bocaj et al. [29]	Schmeling et al. [40]	Dziak et al. [32]	Sturm et al. [41]	Rahman et al. [37]	Corcoran et al. [31]
**Year**	2021	2021	2020	2021	2022	2020	2018	2019
**Qualsyst Score**	83%	71%	70%	96%	75%	100%	86%	67%
**Problem addressed**	Welfare	Wildlife monitoring	Welfare	Welfare	Wildlife monitoring	Increase production	Welfare	Wildlife monitoring
**Fusion application**	Health detection	Activity detection	Activity detection	Activity detection	Individuals recognition	Health detection	Activity detection	Individuals recognition
**Target species**	Pigs	Felines	- Horses - Goats	Cows	- Felines - Birds	Cows	Cows	Koalas
**Animal interface**	-	-	Collar	Collar	-	Ear-tag	-Collar - Ear-tag - Halter	Collar
**Postures**	-	Standing	-	- Standing - Lying	- Standing - Lying on the side	- Walking - Running - Flying postures	- Stationary - Non-Stationary - Lying on belly	Standing
**Activities**	-	- Walking - Running	- Galloping - Walking - Running - Trotting - Feeding/eating - Grassing - Walking (with rider)	- Normal - Walking - Feeding/eating - Resting	- Walking - Running - Trotting - Flying	Ruminating	- Grassing - Ruminating	-
**Participants**	10	-	11	7–11	-	671	-	48
**Sensors**/**Sampling rate (Hz, FPS)**	Visual spectrum cameras	Visual spectrum cameras (30 FPS)	- Accelerometer (100 Hz) - Magnetometer (12 Hz) - Gyroscope (100 Hz)	- Accelerometer - Magnetometer - Gyroscope - Visual spectrum cameras (60 FPS)	- Visual spectrum cameras - Infrared thermal camera	Accelerometer (10 Hz)	Accelerometer (30 Hz)	Infrared thermal camera (9 Hz)
**Feature extraction**	- Histogram-oriented gradients - Local binary patterns - ML models (LSTM-models, CNN-models, etc.)	ML models (LSTM-models, CNN-models, etc.)	ML models (LSTM-models, CNN-models, etc.)	ML models (LSTM-models, CNN-models, etc.)	ML models (LSTM-models, CNN-models, etc.)	ML models (LSTM-models, CNN-models, etc.)	-	ML models (LSTM-models, CNN-models, etc.)
**Feature type**	Spatial	Spatiotemporal	-	- Spatiotemporal - Motion	- Spatiotemporal - Motion	- Spatial - Statistical	- Statistical - Frequency domain	-
**Data alignment**	Same datasize	Same datasize	Same datasize	-	Downsampling	Timestamps	Timestamps	Same datasize
**Machine learning algorithms**	- Convolutional Neural Networks - Bayesian-CNN	- VGG - LSTMs	Convolutional Neural Networks	- SVM - Naive Bayes - Random forest	- YOLO - FASTER RCNN	- Naive Bayes - Nearest centroid classification	Random forest	- Convolutional Neural Networks - YOLO - FASTER RCNN
**Sensor fusion type**	Two or more classifiers	Single fusion algorithm	Single fusion algorithm	Multimodal switching	Single fusion algorithm	Feature level fusion	Mixing	Two or more classifiers
**Performance metrics**	- Accuracy (98.12%) - Precision	Accuracy (92%)	- Accuracy (97.42%) - F1-score (+84%)	Accuracy (87.3%)	Accuracy (94%)	- Accuracy (72.58%) - Sensibility/Recall (66.98%) - Precision (32.27%) - F1-score (43.56%) - Mattews correlation (31.55%) - Youdens index (40.61%)	F1-score (93.2%)	Probability of detection (87%) Precision (49%)

## Data Availability

Not applicable.
